# Assessing the safety and suitability of using silver vine as an olfactory enrichment for cats

**DOI:** 10.1016/j.isci.2023.107848

**Published:** 2023-09-07

**Authors:** Reiko Uenoyama, Sae Ooka, Tamako Miyazaki, Hiroki Mizumoto, Toshio Nishikawa, Jane L. Hurst, Masao Miyazaki

**Affiliations:** 1 Department of Bioresources Science, The United Graduate School of Agricultural Sciences, Iwate University, 3-18-8 Ueda, Morioka, Iwate 020-8550, Japan; 2 Laboratory of Organic Chemistry, Graduate School of Bioagricultural Sciences, Nagoya University, Chikusa, Nagoya 464-8601, Japan; 3 Cooperative Department of Veterinary Medicine, Faculty of Agriculture, Iwate University, 3-18-8 Ueda, Morioka, Iwate 020-8550, Japan; 4 Mammalian Behaviour & Evolution Group, Institute of Infection, Veterinary and Ecological Sciences, University of Liverpool, Leahurst Campus, Neston CH64 7TE, UK; 5 Department of Biological Chemistry and Food Sciences, Faculty of Agriculture, Iwate University, 3-18-8 Ueda, Morioka, Iwate 020-8550, Japan

**Keywords:** Animals, Toxicology, Phytochemistry

## Abstract

Olfactory enrichment is a strategy that can improve welfare among animals managed in captivity, such as household domestic cats. Catnip (*Nepeta cataria*) and silver vine (*Actinidia polygama*) that produce iridoids are used as olfactory enrichments for cats, but little is known about the safety or the best plant resources to use that maximize positive cat responses. We report physiological effects and suitable harvest and drying methods for using silver vine as olfactory enrichment. Continuous exposure of cats to silver vine showed no hallmarks of addictive behavior, while blood indicators of stress and hepatic or renal injury showed no increase in cats stimulated with it. Drying the leaves changed the iridoid profile, enhancing the feline response. In conclusion, dried silver vine leaves are the most suitable resource for developing olfactory enrichment that maximizes feline typical response, which would not result in dependence, stress, or toxicity to the liver or kidneys in cats.

## Highlights

• The feline iridoid response shows no hallmark of addiction or stress
• Repeated exposure to silver vine does not affect biomarkers of hepatic or renal injury
• Cats show a strong response to dried silver vine leaves with complex iridoids
• Dried silver vine leaves are useful for olfactory enrichment in cats

## Graphical abstract

**Figure:**
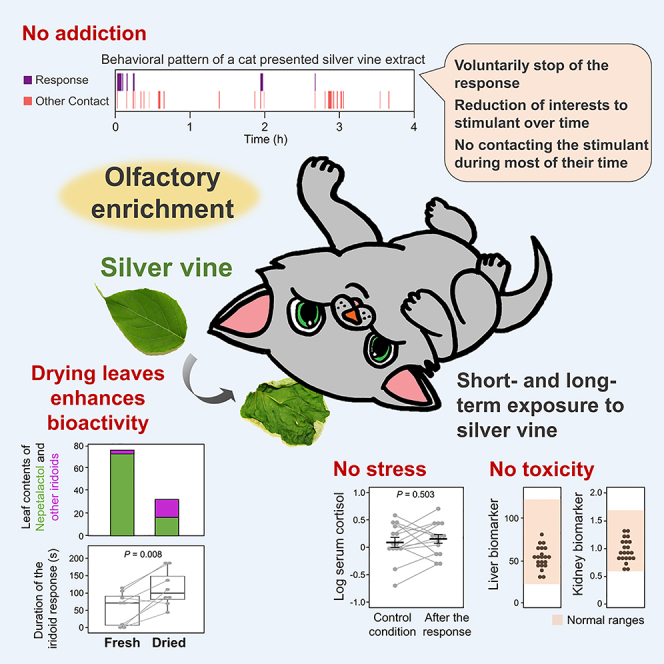

## Introduction

The domestic cat (*Felis silvestris catus*) is one of the most popular companion animals worldwide, with numbers estimated at over 300 million.^1^ The number of cats that are kept exclusively indoors has been increasing because of urbanization.^2^ This housing strategy avoids risks of injury or infectious diseases from the outside environment and helps to maintain health and longevity of cats.^3^ Additionally, the strategy would reduce the impact of free-roaming cats on native wildlife that they prey on.^4^^,^^5^ However, an indoor lifestyle increases the risk of boredom and frustration in cats.^2^ To minimize the negative impacts of keeping cats indoors, recommended enrichment often includes a variety of toys, such as balls, scratchers, stuffed animals, wire-based toys, fishing rod toys, and light-beam pointer games.^6^^,^^7^^,^^8^ Some of these cat toys are supplemented with odors for olfactory enrichment, which play an important role in the welfare of cats.^7^^,^^9^^,^^10^^,^^11^^,^^12^

Domestic cats are olfactory-oriented animals^13^ that commonly use volatile organic compounds emitted from their bodies or from excretions for intraspecific communication.^14^^,^^15^^,^^16^ Many commercial enrichment products are designed to stimulate their olfaction, including synthetic pheromones such as Feliway (Ceva Animal Health) that mimic the chemical components of secretions from the feline cheek and perioral glands.^7^^,^^10^ Some plants, such as silver vine (*Actinidia polygama*) and catnip (*Nepeta cataria*), are also often used as olfactory enrichments for cats.^9^^,^^17^^,^^18^ These plants induce a characteristic behavioral response in cats: licking and chewing, rubbing the face and head against the plants, and rolling over are typically observed.^17^^,^^19^^,^^20^^,^^21^ While veterinarians and pet owners in countries other than East Asia may not be familiar with the silver vine plant, which has a natural distribution restricted to this region, cats exhibit a more intense response to silver vine than catnip.^22^ This feline response is induced through the olfactory system by plant secondary metabolites that are categorized as iridoids, such as iridomyrmecin, isoiridomyrmecin, dihydronepetalactone, isodihydronepetalactone, and nepetalactol in silver vine, and nepetalactone in catnip.^23^^,^^24^^,^^25^^,^^26^^,^^27^ Many pet enrichments prepared from these plants are commercially available worldwide, including dried plant materials, powders, extracted liquids, and soft toys stuffed with plant material.

In a previous study, we reported that the μ-opioid system, which is associated with euphoria and rewarding effects in humans,^28^ is activated in response to specific plant iridoids (the iridoid response) by increasing the β-endorphin levels in cats.^27^ Silver vine and catnip are generally considered to be nontoxic based on their absence from lists of animal poisons, there are no reports supporting their addictive potential,^17^^,^^22^^,^^29^ and no marked physiological or histological effects have been observed within 96 h after ingestion of nepetalactone.^30^ These observations strongly suggest that using silver vine and catnip as enrichments has the potential to improve the well-being of household cats without any negative effects. However, there is a lack of the evidence demonstrating their safety without addictive, toxic, or negative effect by scientific data from statistical behavioral analyses with several cats and blood tests. Therefore, some pet owners are concerned that these plants may be addictive to cats, similar to the exogenous opioid morphine.^31^ In addition, there is a lack of information on the tissue distribution, seasonal variation, and the effect of drying plant materials on the amount and composition of bioactive compounds by analyses using fresh silver vine plants, all of which are important for producing effective olfactory enrichment products for cats. We have shown that the iridoid profile is an important factor in evoking the feline response.^32^ Cats prefer a 1:1 ratio of nepetalactol:other iridoids (iridomyrmecin, isoiridomyrmecin, dihydronepetalactone, and isodihydronepetalactone), which is the iridoid profile observed when silver vine leaves are physically damaged, over the 9:1 ratio produced by fresh intact leaves harvested during the Japanese summer (July). The distribution of silver vine is mainly limited to mountainous areas (not higher than 500 m above sea level) of East Asia, such as Japan, China, and Korea.^33^ Other *Actinidia* species could be better candidates for commercial production if they also produce bioactive iridoids that are effective for cat enrichment. For example, *Actinidia deliciosa* is grown as a commercial crop for people in New Zealand, Italy, Chile, and many other countries,^34^^,^^35^ while *Actinidia arguta* and *Actinidia kolomikta* have greater cold tolerance than silver vine.^33^

This is the first study to address the safety of silver vine by a statistical analysis of behavioral data and blood tests and to examine suitable harvest and drying methods for production of olfactory enrichments using fresh plant samples across different seasons, *Actinidia* species, and plant parts. First, we examined effects of silver vine on cats with single and long-term exposure to iridoid-containing plants and their extracts. We assessed whether cats showed any indication of addiction in response to prolonged exposure to silver vine, or evidence of stress immediately after the response to silver vine through elevated serum cortisol or glucose levels. We also looked for abnormal values of serum biomarkers on liver or kidney diseases in cats with a full history of exposure to iridoids over several years. After confirming that silver vine had no negative effects on cats, we evaluated the concentration of each type of bioactive iridoids (nepetalactol, isodihydronepetalactone, isoiridomyrmecin, and dihydronepetalactone) in different parts of silver vine and other *Actinidia* species, across the potential harvest season and in response to drying. While a number of different iridoids, such as *trans*-dihydronepetalactone, *trans*-isodihydronepetalacone, neonepetalactone, isoneonepetalactone, dihydroactinidiolide, and actinidine, are also present,^17^^,^^24^^,^^36^^,^^37^ a mixture of the major iridoids tested here is sufficient to induce a full behavioral response of similar intensity to damaged leaves.^32^ Our findings clarify the safety of an olfactory enrichment made from silver vine and provide a window into suitable harvest and drying methods in the development of iridoid-based products for cats.

## Results

### Silver vine is not addictive to cats

**Figure 1:**
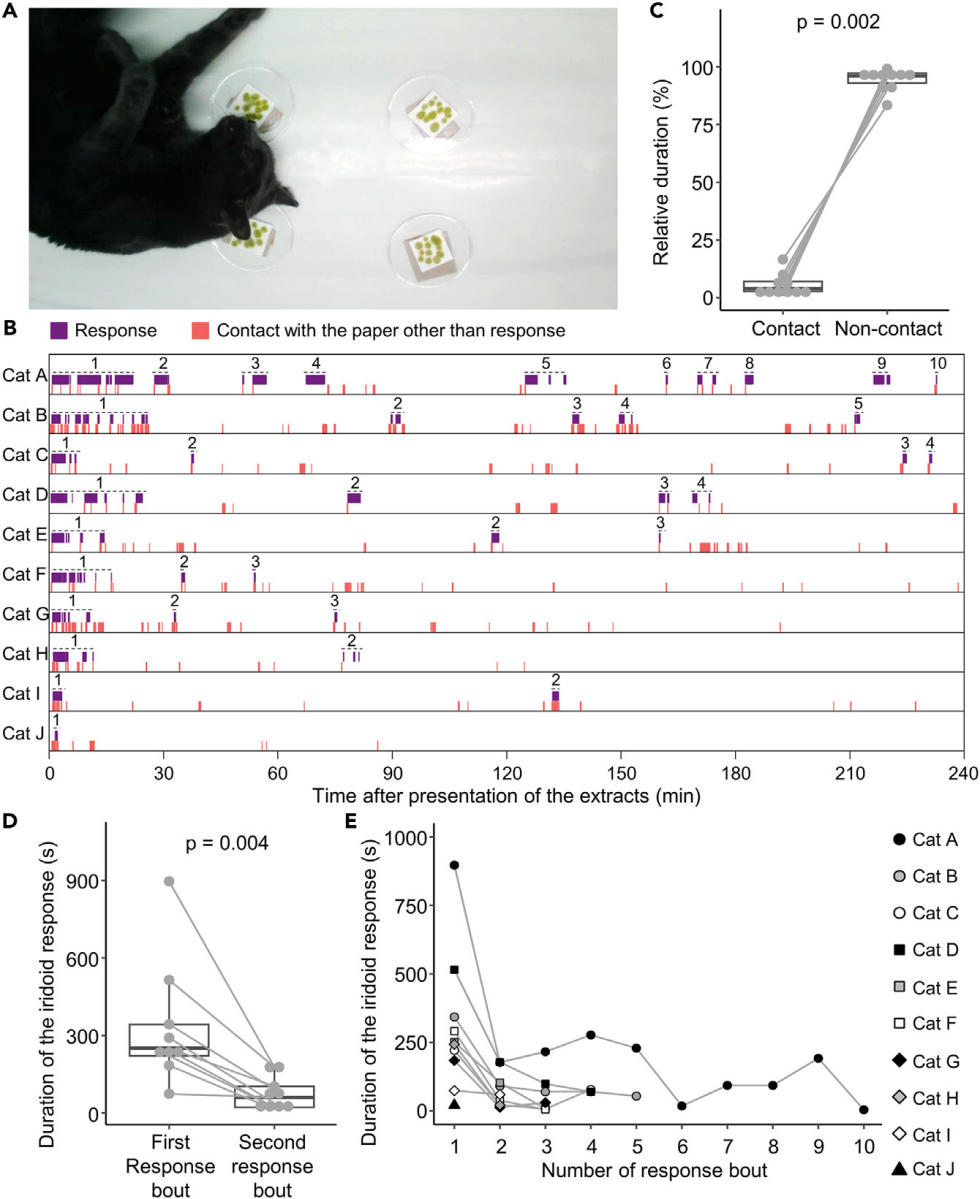
Behavioral pattern of cats exposed to silver vine extracts continuously for 4 h (A) Part of the iridoid response of a cat. A cat is rubbing its face against a piece of paper soaked with silver vine extract in a bioassay. (B) Behavioral patterns of subject cats (*N* = 10). Behaviors were classified into the iridoid response (licking, chewing, rubbing against the stimulus paper, and rolling on the paper; purple) and contact with the paper other than the iridoid response behaviors (sniffing, touching, etc.; pink). Each response bout was defined to start from the first iridoid response behavior and end when response behavior was then followed by > 5 min with no further contact with the paper (shown as a number and a dashed line for each cat). (C) Relative duration of contact with the extract (response, sniffing, and/or touching) and non-contact behaviors (walking, sitting, grooming, lying, and/or sleeping) (*N* = 10 cats). (D) Duration of first and second response bouts (*N* = 9 cats except for Cat J which had only one response bout). (E) The duration of each response bout in sequence for individual cats (separate lines and symbols) (*N* = 10 cats). (C and E) Box and whisker plots show the median, interquartile range, the first data point within the 1.5 interquartile, and individual values. Points connected by lines indicate data from the same individual. p-values are from a two-tailed Wilcoxon matched-pair test. See also Figure S1.

Common hallmarks of substance dependence in humans and in other mammalian groups, including other primates and rodents, are an extremely high motivation to take the substance and difficulty in stopping use.^38^^,^^39^ We hypothesized that if silver vine is addictive for cats, they will not voluntarily stop responding to the plant during prolonged free access, interest will not reduce over time and they will choose to spend most of their time contacting the plant. To test the hypothesis, we presented 10 subject cats with four pieces of filter paper soaked with silver vine extract for a continuous period of 4 h (Figure 1A). Each bout of the typical iridoid response to silver vine (rubbing, rolling, licking, or chewing) was assessed to have ended when these behaviors were followed by over 5 min without further contact with the stimulus papers (see STAR methods for more detail). The number of response bouts ranged from 1 to 10 among the cats, with a median of 3 bouts (Figure 1B). The relative amount of time spent in contact with the extract (including sniffing and touching as well as the more specific iridoid response) was substantially less than that of other behaviors that were not directed to the extract, including walking, sitting, grooming, lying, and sleeping (Figure 1C; median time in contact 4%, in other behaviors 96%; p = 0.002). Further, the duration of the iridoid response was lower during the second response bout compared to the first in all cats (Figure 1D; p = 0.004), and remained short in subsequent responses (Figure 1E). No cat depleted all four pieces of paper containing the extract by licking and chewing, indicating that the decline of response was not caused by a loss of bioactive iridoids. In conclusion, this feline response did not match the definition of substance dependence.

### Stimulation with silver vine does not increase some physiological stress indicators

**Figure 2:**
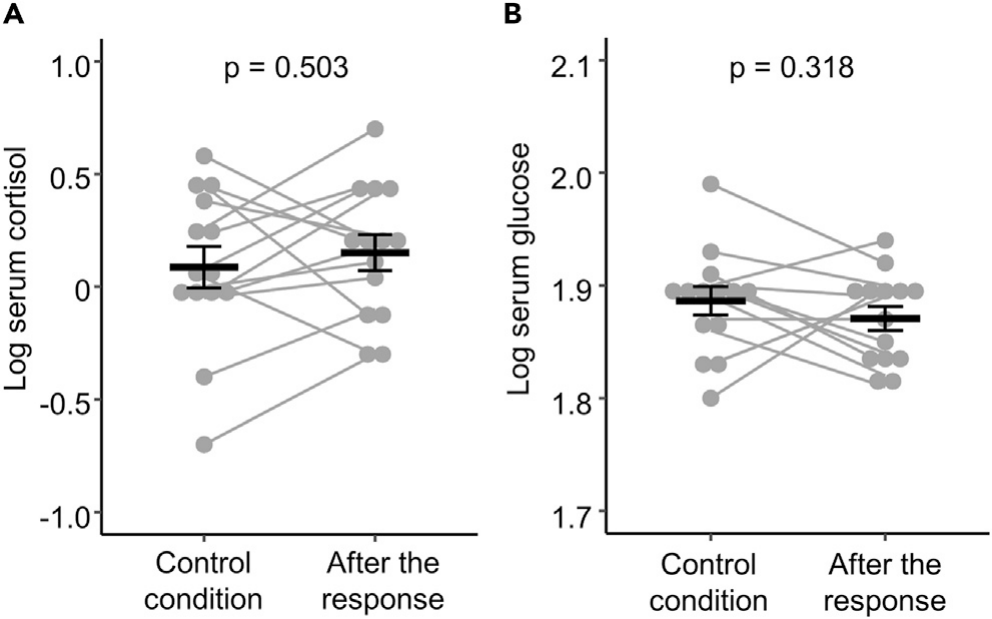
Stress indicators in cats responding to silver vine extract (A and B) Serum concentrations of cortisol (A) and glucose (B) under control conditions and immediately after responding to silver vine extract (*N* = 14 cats). Points connected by lines indicate data from the same individual. Black horizontal lines indicate mean and standard errors. p-values are from paired t-tests (data log-transformed to meet assumptions of parametric analysis).

Transient increases in serum cortisol and glucose levels that are not associated with feeding but with other stimuli in the environment are widely recognized as indicators of stress in animals.^40^^,^^41^^,^^42^^,^^43^ To test whether stimulation by silver vine increases these indicators in cats, we compared serum concentrations of cortisol and glucose in 14 cats between control conditions and after response to silver vine extract. There was no indication of any change in blood levels of cortisol and glucose in response to the extract (Figure 2A: cortisol, p = 0.503; Figure 2B: glucose, p = 0.318).

### Long-term exposure to iridoids has little effect on the liver and kidneys

Next, we examined the effect of long-term exposure to iridoids (up to 3.2 years) on feline liver and kidney function using the serum biomarkers alanine aminotransferase (ALT), which increases with hepatocyte injury,^44^ and creatinine which increases with declining renal function.^45^ Renal diseases are common in elderly cats, with 31% of cats over 9 years that are perceived to be healthy found to have chronic kidney diseases with azotemia when tested.^46^ Here, we evaluated 21 blood test data in total from 13 subject cats under 9 years that had fully recorded histories of exposure to iridoids in the laboratory. Subjects had experienced 0–56 iridoid responses (median: 26) over a period of 11 to 1,176 days (median: 569 days; see Supplemental dataset). All values of ALT and serum creatinine in the subjects were within normal ranges for cats (ALT: 27–124 U/L; serum creatinine: 0.60–1.59 mg/dL). Neither ALT nor serum creatinine levels were influenced by the total number of responses (Table 1; ALT: p = 0.387; serum creatinine: p = 0.861), or the number of days over which cats experienced the iridoid response (ALT: 0.859; serum creatinine: p = 0.135). Furthermore, we also confirmed normal ranges of serum symmetric dimethylarginine (SDMA, mean ± standard error: 10.3 ± 0.55 μg/dL, Table S1) in subject cats, which is a more sensitive biomarker for renal dysfunction than serum creatinine.^47^ Thus, we found no evidence that repeated or long-term exposure to silver vine caused harmful hepatic or renal effects over the 3 year period that we monitored.

**Table 1:** Linear mixed effects model: estimates of fixed effects with log serum ALT and creatinine levels as dependent variables

	Estimate	Standard error	Degree of freedom	*t*	*p*
ALT					
Intercept	1.663	0.047	17.940	35.280	<0.001
Total number of iridoid responses	0.002	0.002	17.850	0.886	0.387
Days over which response experienced	<0.001	<0.001	17.160	0.180	0.859
Serum creatinine					
Intercept	−0.099	0.040	17.960	−2.460	0.024
Total number of iridoid responses	<0.001	0.002	17.334	0.177	0.861
Days over which response experienced	<0.001	<0.001	14.314	1.585	0.135

Note: 21 blood test data in total from 13 subject cats were evaluated. See also Table S1.

### The iridoid content of silver vine varies between plant parts

**Figure 3:**
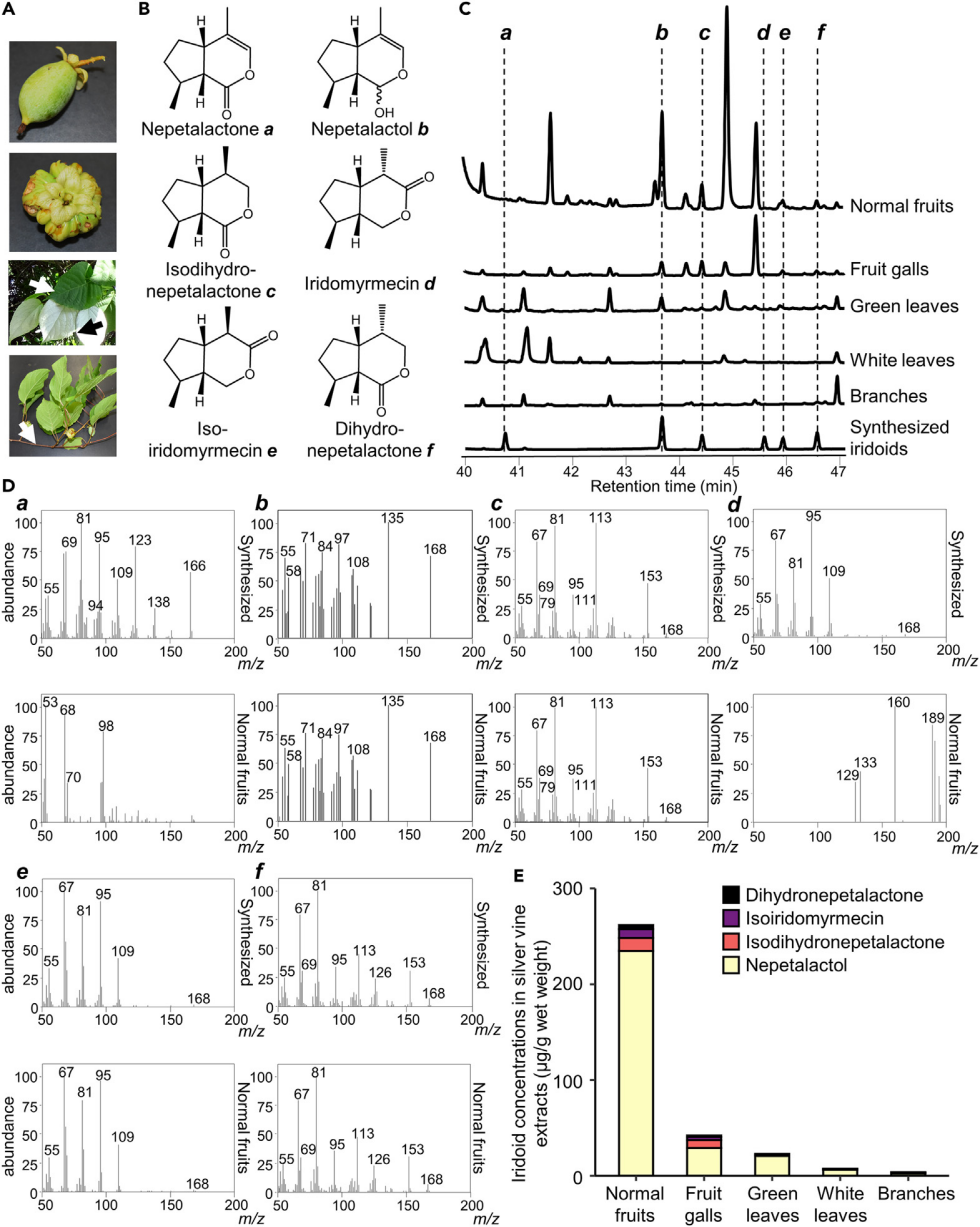
Amount of iridoids extracted from different parts of silver vine (A) Images of a normal fruit, a fruit gall, green (white arrow) and white (black arrow) leaves, and branches (white arrow) of silver vine, harvested in August, from top to bottom. Fruit galls are produced by the parasite midge *Pseudasphondylia matatabi*.^48^^,^^49^ (B) Chemical structures of six iridoids: nepetalactone (*a*), nepetalactol (*b*), isodihydronepetalactone (*c*), iridomyrmecin (*d*), isoiridomyrmecin (*e*), and dihydronepetalactone (*f*). (C) GC/MS total ion chromatograms of extracts of normal fruits, fruit galls, green leaves, white leaves, and branches sampled in early August, and of synthesized iridoids. (D) Mass spectra of the synthesized iridoids and matching peaks detected at the same retention time as the synthesized iridoids in normal fruits. (E) Bars show the concentration of dihydronepetalactone (black), isoiridomyrmecin (purple), isodihydronepetalactone (pink), and nepetalactol (yellow) for each plant part extract.

As silver vine provides an attractive stimulant for cats with no evidence to suggest a risk of addiction, stress, or hepatic or renal injury, next we turned to investigate factors that might influence the harvesting of silver vine to produce an effective cat olfactory stimulant. First, we examined the concentration of bioactive iridoids to cats (specifically: nepetalactol, iridomyrmecin, isoiridomyrmecin, dihydronepetalactone, and isodihydronepetalactone) in different parts of silver vine harvested from a wild population in early August when fruits began to grow (Figure 3A). Comparisons of GC/MS mass spectra between natural compounds in normal fruits and synthesized iridoids confirmed the presence of nepetalactol, isodihydronepetalactone, isoiridomyrmecin, and dihydronepetalactone in natural normal fruits, but not nepetalactone or iridomyrmecin (Figures 3B–3D). While the nepetalactol peak was detectable in all parts of the plants tested, no plant part contained nepetalactone or iridomyrmecin. Isodihydronepetalactone was detectable in all plant parts except for white leaves. Isoiridomyrmecin and dihydronepetalactone were detectable in normal fruits, fruit galls, and green leaves. Normal fruits contained a substantially higher level of the four iridoids combined, at approximately 6-fold that in fruit galls and 11-fold that in green leaves (Figure 3E). The total iridoid content of green leaves was about 3-fold higher than that in white leaves, while branches had the lowest content of total iridoids. Nepetalactol accounted for more than 85% of the total iridoids in green leaves (90.8 ± 0.3%), white leaves (86.9 ± 1.9%), and normal fruits (89.7 ± 0.3%). Fruit galls and branches had relatively higher proportions of iridoids other than nepetalactol (dihydronepetalactone, isoiridomyrmecin, and isodihydronepetalactone), accounting for 30.9 ± 0.4% and 31.1 ± 1.1%, respectively.

### Seasonal changes in silver vine leaf iridoids

**Figure 4:**
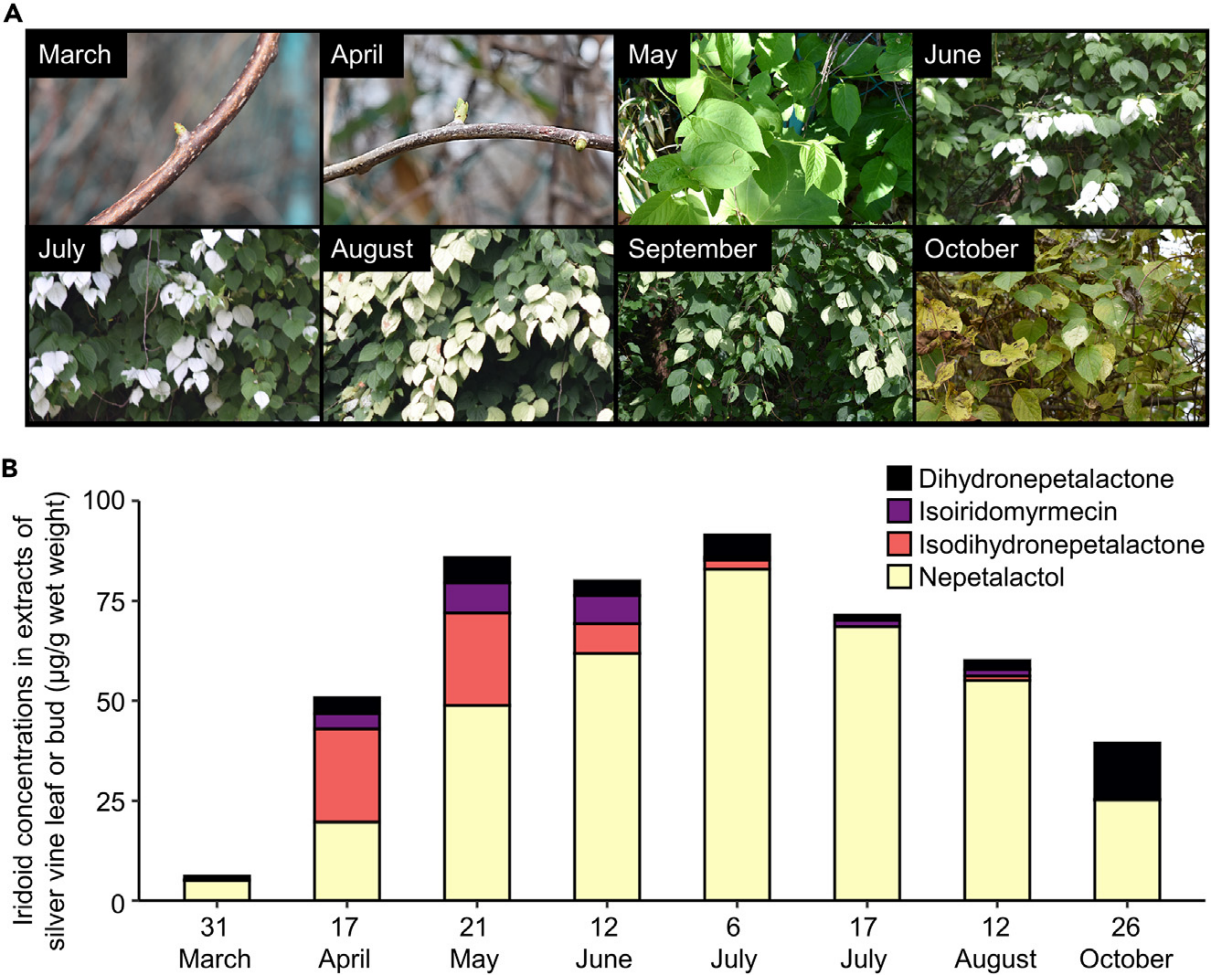
Seasonal variation in the iridoid concentration and profile of silver vine green leaves (A) Images of silver vine buds and leaves harvested from a wild population during March to October 2020. (B) Concentration of each type of iridoid in extracts from fresh green leaves harvested from 31 March to 26 October: dihydronepetalactone (black), isoiridomyrmecin (purple), isodihydronepetalactone (pink), and nepetalactol (yellow).

Although fruits have the highest iridoid content (Figure 3E), fruits are available for a much shorter season (July–August) than the green leaves of silver vine (March–October, Figure 4A). To examine seasonal effects that might influence the optimal harvesting of silver vine for olfactory enrichment products, we analyzed the iridoid content and profile of green leaves or buds of silver vine sampled at 8-time points from March to October. Samples collected between May and August had higher levels of total iridoids (>60 μg/g wet weight) than those collected during early spring (March and April) or fall (October) (Figure 4B). The amount of nepetalactol, which was generally the dominant iridoid in leaves, increased from March to early July and then decreased until October. The highest proportion of iridoids other than nepetalactol was 61.1% ± 0.7% of total iridoids in the buds harvested in April.

### Effects of drying silver vine leaves

**Figure 5:**
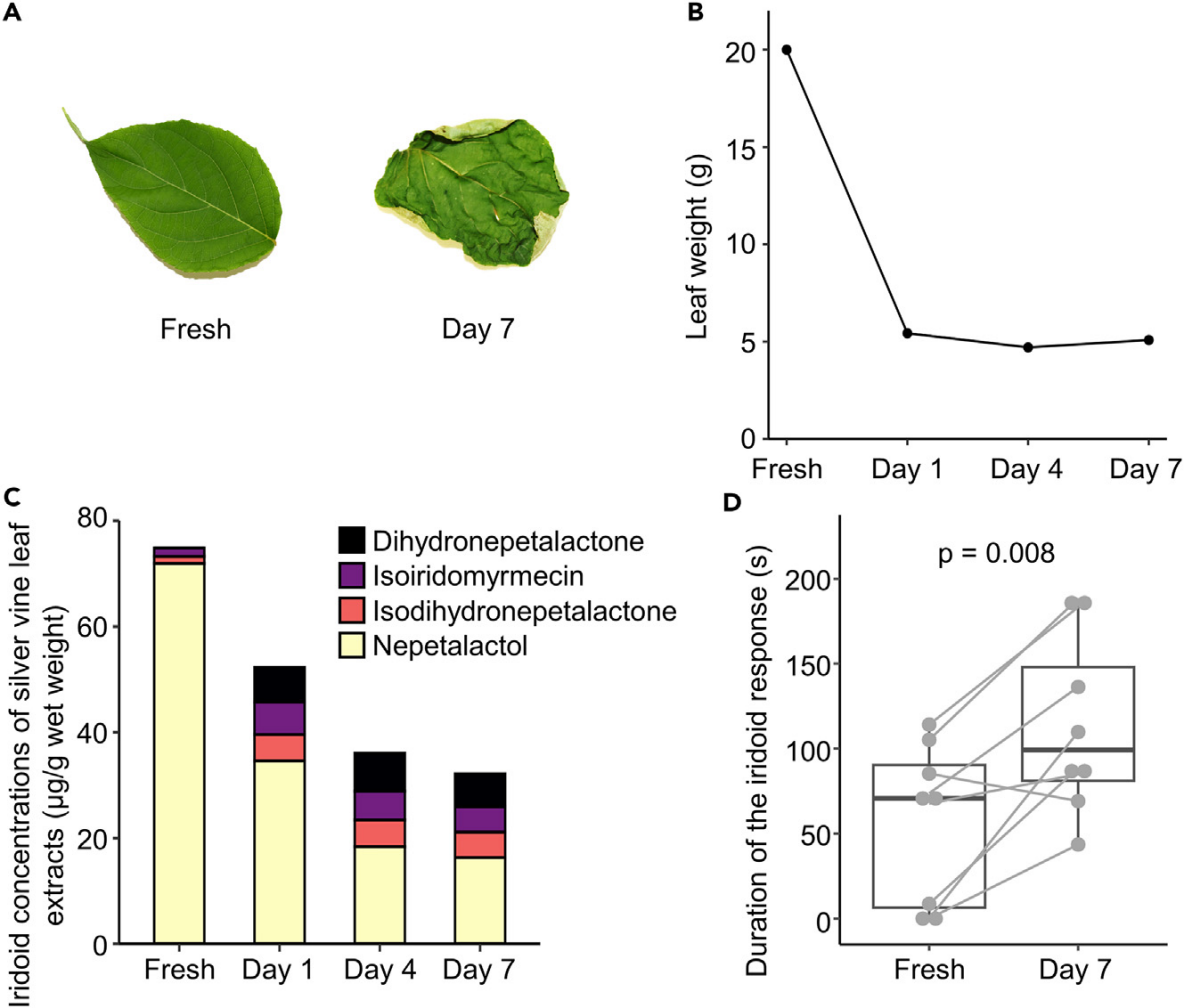
Effects of drying silver vine green leaves on iridoid concentrations and responses of cats (A) Images of silver vine leaves before (Fresh) and after 7 days drying (Day 7) under atmospheric conditions. Fresh leaves (initially 20 g wet weight) harvested in August were used for this experiment. (B) Weights of silver vine leaves before and 1, 4, and 7 days after drying. (C) Temporal changes in the concentrations of iridoids in leaf extracts before and 1, 4, and 7 days after drying: dihydronepetalactone (black), isoiridomyrmecin (purple), isodihydronepetalactone (pink), and nepetalactol (yellow). (D) Duration of the iridoid response (licking, chewing, rubbing, and rolling) to extracts of silver vine green leaves before and 7 days after drying in cats (*N* = 8). Box and whisker plots show the median, interquartile range, first data point within 1.5 interquartile ranges, and individual values. Points connected by lines indicate data from the same individual. p-value is from a two-tailed Wilcoxon matched-pair test.

While dried silver vine leaves are commonly used as olfactory enrichment for cats,^17^^,^^22^ little is known about the effect of drying on the amount and profile of iridoids and bioactivity. We examined temporal changes in the amount and profile of iridoids in green silver vine leaves sampled in August and dried for 7 days under atmospheric conditions (Figure 5A). Leaf weight decreased to approximately 25% of the fresh sample after one day of drying due to the loss of water, and then remained stable (Figure 5B). Total iridoid concentration was highest in fresh leaf samples and decreased gradually with drying to approximately half that of the fresh sample after 7 days (Figure 5C). Importantly, the iridoid profiles changed with drying, similar to changes observed in leaves physically damaged by crumpling and tearing.^32^ Drying for 1 to 7 days increased the total amount of iridoids other than nepetalactol in the leaves (Figure 5C). While dihydronepetalactone, isoiridomyrmecin, and isodihydronepetalactone made up 3.9 ± 0.8% of total iridoids in fresh leaves, this increased to 49.3 ± 0.6% on day 7. Based on this, we compared responsiveness of eight cats toward extracts of fresh green leaves and leaves dried for 7 days in a two-choice test in which both extracts were presented simultaneously (see STAR methods for more detail). The duration of response was more prolonged toward the 7 days-dried extract than toward the fresh leaf extract (Figure 5D; exact p = 0.008). In conclusion, drying silver vine leaves decreased the total amount of iridoids but changed the iridoid profile. This induced a more prolonged response in cats compared to their response to fresh leaves.

### Some Actinidia plants accumulate iridoids in roots rather than leaves

**Figure 6:**
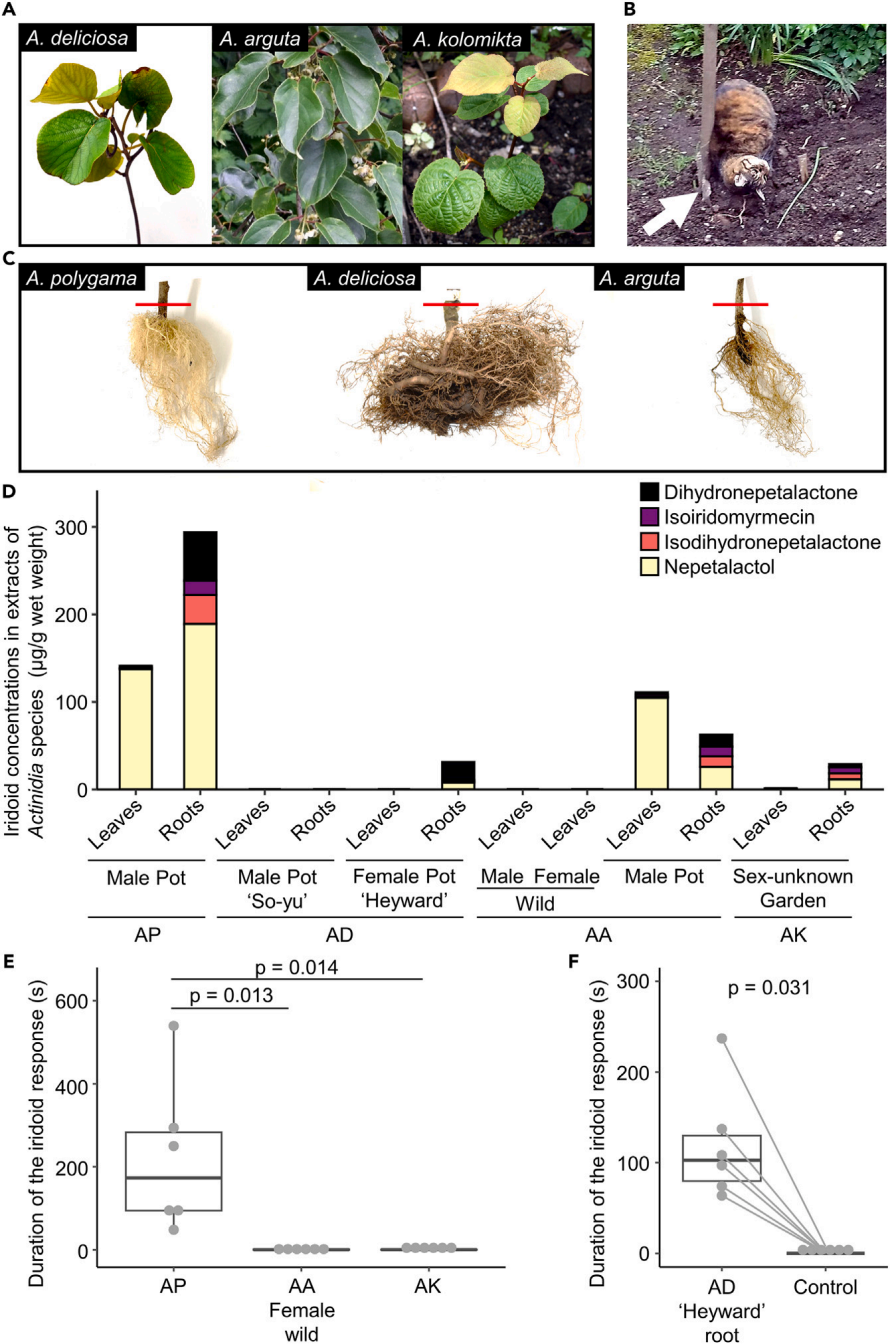
Comparison of iridoid concentrations and bioactivities to cats in different *Actinidia* species (A) Leaf images of *A. deliciosa* (AD), *A. arguta* (AA), and *A. kolomikta* (AK). (B) A cat rubbing its head against *A. deliciosa* roots. White arrow indicates the roots. (C) Root images of silver vine silver vine (*A. polygama*; AP), AD, and AA. The tissues below red lines that show the ground were sampled as roots. (D) Iridoid concentrations in extracts of fresh leaves and roots of AP, AD, AA, and AK: dihydronepetalactone (black), isoiridomyrmecin (purple), isodihydronepetalactone (pink), and nepetalactol (yellow). (E) Duration of the iridoid response (licking, chewing, rubbing, and rolling) to leaf extracts prepared from AP, AD, AA, and AK in cats (*N* = 6). p-values are from Steel–Dwass post hoc tests. (F) Duration of the iridoid response toward root extract from AD and a solvent control in six cats (*N* = 6). p-value is from a two-tailed Wilcoxon matched-pair test. See Video S1.

Cats are occasionally attracted to *A. deliciosa* and *A. arguta*, and rub their faces and heads against the roots (Figures 6A, 6B, and 6C). However, there are very limited studies on behavior toward *Actinidia* species other than silver vine or chemical analyses of other species. As these plants might be additional sources or possible replacements for silver vine, we examined the amount and profile of iridoids in the green leaves and roots of these species. Little to no bioactive iridoids (<3 μg/g wet leaf weight) were detected in leaves of *A. deliciosa* or *A. kolomikta* (sex-unknown *A. kolomikta* from a private garden: isodihydronepetalactone 1.4 ± 0.1 μg/g), or from female *A. arguta* (from a wild population: isoiridomyrmecin 2.1 ± 0.02 μg/g), although leaves collected from male *A. arguta* potted plants contained much higher levels (total iridoids: 111.1 ± 3.0 μg/g) (Figure 6D). By contrast, the roots of ‘Hayward’ female *A. deliciosa* and male *A. arguta* potted plants, and sex-unknown *A. kolomikta* from a garden, contained more than 30 μg/g total iridoids. This was still substantially less than the roots of male silver vine (*A. polygama*) which contained 293.7 ± 6.0 μg/g total iridoids.

As relatively low levels of a single iridoid were detected in leaves of wild *A. arguta* and *A. kolomikta*, we evaluated whether this was sufficient to stimulate the iridoid response. In separate tests, six subject cats were tested with green leaves (5 g aliquot) from *A. arguta* (female from a wild population) and *A. kolomikta* (sex-unknown from the garden), and silver vine as a positive control. All cats responded almost exclusively to silver vine (Figure 6E; Friedman test, χ^2^(2) = 9.00, p = 0.008). Finally, the cats were tested with 100 μL root extract from ‘Hayward’ female *A. deliciosa*, which contained approximately 3 μg of mixed iridoids in total. As expected for this level of iridoids, cats showed a more prolonged response to the root extract than to a solvent control (p = 0.031; Figure 6F and Video S1).

Video S1. The iridoid response toward root extract from *Actinidia deliciosa* in three cats, related to Figure 6F

## Discussion

Silver vine and catnip plants are used as olfactory enrichments for cats, but their safety and the best plant resources to use that maximize positive cat responses had not been thoroughly studied. Thus, the present study provides an in-depth examination of their safety determined by both behavioral analyses and blood tests, and establishes suitable harvest and drying methods for using silver vine as olfactory enrichment. We found no scientific evidence to suggest that silver vine is an addictive substance for cats, nor that exposure stimulates a physiological stress response. Cats stopped responding to the plant extract during prolonged free access, they did not choose to spend most of their time contacting the plant, and duration of each response bout was short (median: approximately 90 s). Short-term exposure of cats to the extract did not affect serum stress indicators, cortisol and glucose. Normal values of serum biomarkers for the liver and kidneys among cats exposed to iridoids for at least 3 years suggest that repeated exposure to silver vine iridoids does not cause significant damage to cat liver or kidneys. Given that nepetalactol in silver vine stimulates the μ-opioid reward system in cats^27^ but our study indicates that exposure to silver vine does not appear to cause cats harm, demonstrating that silver vine and other iridoid-producing plants are suitable olfactory enrichments without addictive, toxic, or negative effect for domestic cats.

The present study examined chemical profiles only of a part of iridoids (nepetalactol, iridomyrmecin, isoiridomyrmecin, dihydronepetalactone, and isodihydronepetalactone). This is because bioactivities of each of these compounds are well examined in previous papers from ourselves,^27^^,^^32^ and from other laboratories^24^^,^^25^ as compared to the other iridoids (*trans*-dihydronepetalactone, *trans*-isodihydronepetalactone, neonepetalactone, isoneonepetalactone, dihydroactinidiolide, and actinidine). Our previous data^27^ and Bol et al.^17^ reported that actinidine has little bioactivities for cats and has a chemical structure carrying nitrogen that is markedly different from other iridoids carrying oxygen that have high bioactivity. The evidence for bioactivities of neonepetalactone, isoneonepetalactone, and dihydroactinidiolide is currently weak, because these were tested only on five cats and elicited low response time in cats.^17^ We think that it is required to do more experiments to determine their bioactivities for cats. The related study^17^ did not conduct independent tests of bioactivities (for example, all cats were tested together, stimuli were presented in socks that would pick up scents from prior cat interactions), and compounds were tested at arbitrary amounts without relation to the amount in plants. Therefore, we focused on the 6 iridoids in this study that currently have substantial data confirming bioactivity. This dataset of iridoid profiles is enough to discuss the suitable harvest and drying methods questions addressed in our study.

Optimum harvest and preparation of silver vine requires an understanding of the tissue distribution of plant iridoids, along with seasonal changes and effects of drying on iridoid levels and the impact on feline bioactivity. Iridoid levels were higher in normal fruits and fruit galls than in other plant parts, and were at maximum levels in green leaves from May to August as compared to the other seasons. The iridoid profile changed to a 1:1 ratio of nepetalactol:other iridoids in green leaves after 4–7 days of drying, which induced greater stimulation in cats. As the other *Actinidia* species tested (*A. deliciosa*, *A. arguta*, *A. kolomikta*) generally contained much lower levels of iridoids, although had some iridoids in their roots, they are not likely to provide useful replacements for silver vine. Based on our study, we suggest that dried green leaves of silver vine, or fruits, will provide the most suitable sources of iridoids for cat olfactory enrichment.

Cats showed a gradual loss of interest and spontaneously stopped showing their iridoid response to silver vine over 4 h of exposure, consistent with a lack of addictive response to these plants. This is very interesting because the μ-opioid system is activated by an endogenous opiate β-endorphin, which is involved in the expression of the iridoid response in cats.^27^ Our findings may also contribute to find the potential to stimulate the opioid system without human chemical dependence. Most of the other stimulants that activate the μ-opioid system via an increase in β-endorphin, such as alcohol and Δ^9^-tetrahydrocannabinol (Δ^9^-THC) in marijuana, are addictive.^50^^,^^51^^,^^52^ Morphine, an exogenous opiate used as a painkiller, has the potential to be highly addictive, as tolerance to it develops rapidly.^31^ This discrepancy between iridoids and other addictive compounds might be explained by the finding that iridoids elicit their characteristic response via the olfactory system,^53^ while alcohol, Δ^9^-THC, and morphine activate GABA receptors, cannabinoid receptors, and opioid receptors, respectively, directly via the bloodstream.^54^^,^^55^^,^^56^ In the absence of any negative physiological stress response via activation of the hypothalamus-pituitary-adrenal (HPA) axis, evidenced by lack of variation of serum cortisol and glucose levels, silver vine is likely to contribute positively to cat welfare by stimulating the μ-opioid system without inducing addiction or stress.

As some plants, such as lilies (*Lilium candidum*), can be lethally toxic to the kidneys of cats and cause acute renal failure,^57^ it is important to evaluate the effects of plant metabolites, particularly those with bioactivities, prior to recommending their use for animal enrichment. Blood biochemical tests suggest that silver vine had no major side effects on liver and kidney health in cats that had been frequently exposed over a period of approximately three years. This is supported by previous reports that some Asians have used silver vine as an ingredient in liquor or oriental medicine for pain, inflammation, gout, and rheumatoid arthritis.^58^^,^^59^^,^^60^

The drying of green leaves of silver vine sampled in August caused a similar increase in the levels of iridoids other than nepetalactol (isoiridomyrmecin, isodihydronepetalactone, and dihydronepetalactone) as manually crumpling and tearing the leaves.^32^ As dried silver vine leaves prolonged feline response similar to the response to manually damaged leaves, dried leaves are particularly suitable for use in olfactory enrichment products. The levels of iridoids other than nepetalactol were similar in green leaves dried for 1, 4, or 7 days, suggesting that one day of drying should be sufficient to enhance cat bioactivity while reducing the weight of the plant material. This information will be particularly valuable for preparing plant resources commercially for enrichment products, as it is only necessary to dry leaves at room temperature under atmospheric conditions and no manual crumpling or tearing is required.

Fresh green leaves of silver vine collected in April and May had a natural 1:1 ratio of nepetalactol:other iridoids, a ratio that is most effective for stimulating cats.^32^ However, it can be difficult to distinguish silver vine plants from other climbing vines in wild populations before the unique white leaves of silver vine appear later in the growing season. We propose that green leaves of silver vine cultivated between early June and August and dried for at least 1 day will provide a better resource for the development of products that incorporate this olfactory cat stimulant, both to maximize the bioactivity for cats and minimize the weight of plant material required. On the other hand, normal fruits, which contained the highest contents of total iridoids, and fruit galls, which contained approximately 2:3 nepetalactol:other iridoids ratio, are also candidate resources for cat olfactory enrichment, although fruits have a more limited season (for example, they can be sampled only during late July and early August in the Iwate prefecture). Nepetalactol accounted for over 85% in fresh green leaves of silver vine collected during July and August, which is consistent with our previous studies.^27^^,^^32^ Another group also detected nepetalactol as the iridoid present at the highest amount in silver vine leaves,^61^ while a recent study did not identify nepetalactol in silver vine.^17^ Looking at differences in the methodologies used in these studies, the most likely explanation would be that Bol et al. analyzed only dried plant samples.

*A. deliciosa*, *A. arguta*, and *A. kolomikta* mostly contained iridoids in roots rather than in leaves. The localization of iridoids in the roots is similar to other plants such as *Valeriana officinalis* and *Acalypha indica* that emit iridoids that induce the typical chewing and rolling response in cats from their roots and the medicinal herbs *Castilleja tenuiflora* Benth., and *Triosteum pinnatifidum*.^22^^,^^62^^,^^63^^,^^64^^,^^65^ A previous study detected nepetalactol at moderate levels in *A. arguta* leaves,^61^ but in our analysis this differed between samples from wild populations and potted plants. This suggests that a wide range of *Actinidia* plants and some other plant species that have been reported to contain iridoids possess a biosynthetic iridoid pathway, while the tissue localization of iridoids differs among plant species, varieties, and the growing environment. To obtain a large yield of iridoids sustainably, without the trouble of removing soil from plant samples, we suggest that green leaves and fruits of silver vine will be more suitable resources to develop feline olfactory enrichment products than other *Actinidia* plants that contain iridoids largely in their roots.

Another important finding from our study is that half of 10 subject cats showed repeated bouts of the iridoid response within 30 min (Figure 1B). This contrasts with a previous report that cats are non-responsive to catnip for one or more hours after showing a typical response.^53^ However, this was based on citation of another study^20^ in which we could not find the cited evidence. More importantly, our results also demonstrated that numbers of response bouts are highly variable among subjects. A previous report suggested that this response is inherited as an autosomal dominant trait in cat populations that contain 30% negative responders to catnip.^21^^,^^22^ Our findings add to this by showing that positive responder cats vary substantially in their responsiveness to iridoids among cats may be more complex.

In conclusion, our study provides valuable information on the safety of silver vine as an olfactory enrichment for household cats, together with the most suitable sources for harvest and drying conditions. Toys that incorporate this plant would not result in dependence, stress, or toxicity to the liver or kidneys. The green leaves of silver vine provide the best source because these are easy to harvest over a long season and, when dried, they contain effective levels of iridoids for strong bioactivity. Drying enhances the effects on cats, reduces the weight of plant material, and allows for easy storage at room temperature. Silver vine fruits also provide another rich source. This will help suppliers of enrichment products, veterinarians, and pet owners to become aware of the potential of using silver vine as an effective tool to improve the quality of life for household cats. Moreover, improving the indoor lifestyle for cats through using this enrichment may help to encourage owners to keep cats indoors in situations where conservation of native wildlife prey is a significant problem.

### Limitations of the study

We found that numbers of response bouts are highly variable among cats in experiments presenting the silver vine extract over 4 h, but could not explain the cause for the individual difference in them. In this study, we observed that the number of response bouts tended to be similar among related cats (four or more bouts in cats A–D and three bouts in cats F and G, Figure S1) and decreased with aging (twice in cats H and I aged 12 years old) (Figure 1B, Data S1). Variation in response intensity among cats may be attributed to a combination of genetic and aging factors. Further studies, such as bioinformatic genome analysis using cats that differ in their level of responsiveness to iridoid stimuli (including those that do not respond) would help to answer the question. It would also be interesting to examine whether elderly cats may have reduced olfactory sensitivity to odorants including iridoids as has been found in humans and rodents^66^ and to understand more about the “habituation” (reduced responding over time) that cats show to these olfactory stimuli.

The small sample size of plants harvested in a limited area without replication and statistical analysis in our study means that it is hard to conclude whether differences in iridoid content and profiles we found among plant parts, sampling season, fresh and dried leaves, and plant species are generalized. For example, Bol et al. report much lower levels of iridoids in dried fruits compared to the fresh fruits reported here.^22^ Continuous analysis of iridoid contents and profiles in silver vine for several years will be lines of evidence that may support our conclusions in future studies.

## STAR★Methods

### Key resources table

**Table:** 

REAGENT or RESOURCE	SOURCE	IDENTIFIER
**Chemicals, peptides, and recombinant proteins**		

Nepetalactone	Adachi et al.^67^ Schreiber et al.^68^ Uenoyama et al.^27^	CAS: 21651-62-7
Nepetalactol	Adachi et al.^67^ Schreiber et al.^68^	CAS: 109215-55-6
Dihydronepetalactone	Adachi et al.^67^ Schreiber et al.^68^ Uenoyama et al.^27^	CAS: 21950-33-4
Isodihydronepetalactone	Adachi et al.^67^ Schreiber et al.^68^ Uenoyama et al.^27^	CAS: 24190-27-0
Iridomyrmecin	Adachi et al.^67^ Schreiber et al.^68^	CAS: 485-43-8
Isoiridomyrmecin	Adachi et al.^67^ Schreiber et al.^68^	CAS: 107538-14-7
Chloroform	FUJIFILM Wako Pure Chemical	034–02608
Methanol	FUJIFILM Wako Pure Chemical	139–01827
*n*-Hexane	FUJIFILM Wako Pure Chemical	080–03423
L-Type ALT	FUJIFILM Wako Pure Chemical	465–30603
LabAssay ™ Glucose	FUJIFILM Wako Pure Chemical	638–50971
LabAssay ™ Creatinine	FUJIFILM Wako Pure Chemical	636–51011

**Deposited data**		

Raw and analyzed data	This paper	Data S1

**Experimental models: Organisms/strains**		

Domestic cat (*Felis silvestris catus*)	Kitayama Labes Co.,Ltd.	N/A
Silver vine (*Actinidia polygama*; sex-unknown; in wild populations)	A wild population in Morioka, Iwate Prefecture, Japan (coordinate: 39°45′17″ N 141°10′26′ E), another wild population in Morioka (39°45′21″ N 141°08′40'' E), or a wild population in Takizawa, Iwate Prefecture (39°48′16'' N141°06′17'' E)	N/A
Silver vine (male; potted plants)	A plant nursery in Morioka, Japan	N/A
*Actinidia deliciosa* (female; cultivar ‘Heyward’; potted plants)	A plant nursery in Morioka, Japan	N/A
*Actinidia deliciosa* (male; cultivar ‘So-yu’; potted plants)	A plant nursery in Morioka, Japan	N/A
*Actinidia arguta* (male; in wild population)	A wild population in Karumai, Iwate Prefecture (40°16′50″ N 141°23′6'' E)	N/A
*Actinidia arguta* (female; in wild population)	A wild population in Karumai, Iwate Prefecture (40°16′28″ north latitude, 141°23′19'' east longitude)	N/A
*Actinidia arguta* (male; potted plants)	A plant nursery in Morioka, Japan	N/A
*Actinidia kolomikta* (sex-unknown)	A private garden in Morioka, Iwate Prefecture, Japan	N/A

**Software and algorithms**		

SPSS version 29.0.0.0	IBM Corp.	https://www.ibm.com/analytics/spss-statisticssoftware
JMP version 12.0.0	SAS Institute Inc.	https://www.jmp.com/en_us/software/data-analysis-software.html
R version 4.2.2	The R Foundation for Statistical Computing	https://mirrors.bfsu.edu.cn/CRAN/index.html
Behavioral Observation Research Interactive Software (BORIS) version 8.9.11	Olivier Friard and Marco Gamba	http://www.boris.unito.it/pages/download.html
GCMSsolution version 4.53	Shimadzu Co.	https://www.an.shimadzu.co.jp/gcms/support/download/gcms_s/gcms453sp1dl.htm

**Other**		

Filter paper for filtration of plant extracts	Toyo Roshi Kaisha Ltd.	Advantec qualitative no. 2
Rotary evaporator	EYELA	N-1200A
GC/MS	Shimadzu Co.	QP-2010 Ultra
Autosampler	Shimadzu Co.	AOC-20i/s
InertCap Pure-WAX column	GLScience	1010–68162
Cat cage	DCM Co., Ltd.	456063
Digital video camera	Sony	Handycam HDR-CX680
Petri dish (9 cm diameter)	Sansyo Co., Ltd.	36–3407
Filter paper for behavioral assays	Toyo Roshi Kaisha Ltd.	Advantec No. 526

### Resource availability

#### Lead contact

Further information and requests for resources and reagents should be directed to and will be fulfilled by the Lead Contact, Masao Miyazaki (mmasao@iwate-u.ac.jp).

#### Materials availability

All data supporting the synthesised iridoids can be received from the lead contact upon request. The synthesised iridoids used in this study will be made available on request by the lead contact with a completed Materials Transfer Agreement.

### Experimental model and study participant details

#### Animals

Eighteen healthy mixed-breed domestic cats (age 10 months to 12 years; seven intact males, eight intact females, and three spayed females) were used. The cats were experimental animals living at Iwate University. They were maintained at 24°C under a 10 h light:14 h dark photoperiod (lights on at 7:00 a.m.) to keep the females in anoestrus.^67^ The cats were housed in pairs or individually in three-story cages (93 × 63 × 178 cm) with continuous visual, auditory, and olfactory contact with other cats in the room. The cats received dry food twice daily (at 8:30 a.m. and 4:00 p.m.) and had continuous access to drinking water. Veterinarians of Iwate University performed regular health checks, in addition to blood biochemical tests at least once per year. The Animal Research Committee of Iwate University approved all behavioral assays (approval number: A202124). A Data S1 shows which cats participated in the experiments. They also participated in other experiments to study scent communication in cats, wherein they were presented with urine samples of other cats during the same period as this study.

#### Plants

Fresh and undamaged normal fruits, fruit galls, green leaves, white leaves, and branches of silver vine (sex-unknown) were sampled from a wild population in Morioka, Iwate Prefecture, Japan (39°45′17″N 141°10′26″E on 6 August, 2019) for comparison of iridoid contents and profiles between different plant parts. To examine seasonal changes in leaf iridoids, fresh undamaged green leaves of silver vine (sex-unknown) were sampled from another wild population in Morioka (39°45′21″N 141°08′40″E on 31 March, 17 April, 21 May, 12 June, 6 July, 17 July, 12 August, and 26 October, 2020). To compare iridoid contents and profiles among plant species, fresh undamaged green leaves of male silver vine and the roots that were portions in the ground were collected from potted plants purchased at a Japanese plant nursery on 13 September, 2022. Green leaves and roots of *A. deliciosa* (female cultivar ‘Hayward’ and male cultivar ‘So-yu’) were collected from potted plants purchased at a Japanese plant nursery on 1 September, 2022. Green leaves and roots of *A. arguta* were sampled from wild populations in Karumai, Iwate Prefecture (only leaves at 40°16′50″N 141°23′6″E on 18 June, 2021 for males; at 40°16′28″N 141°23′19″E on 18 June, 2021 for females) or collected from potted male plants purchased at a Japanese plant nursery on 13 September, 2022. Green leaves and roots of sex-unknown *A. kolomikta* were harvested from a private garden in Morioka on 7 September, 2022. No permissions are required for *Actinidia* species in Japan, as none of the species are endangered.

#### Chemicals

Chloroform (reagent grade, ≥99.0% purity), methanol (reagent grade, ≥99.8% purity), and *n*-hexane (HPLC grade, ≥96.0% purity) were purchased from Fujifilm Wako Pure Chemical Corp. (Osaka, Japan). Nepetalactone, nepetalactol, iridomyrmecin, isoiridomyrmecin, dihydronepetalactone, and isodihydronepetalactone were synthesised according to previous reports.^68^^,^^69^^,^^70^

### Method details

#### Preparation of organic solvent extracts of fresh plant samples

Plant samples were frozen in liquid nitrogen immediately after harvesting (potted plants), within 30 min after harvesting (silver vine from wild populations and *A. kolomikta* from a garden), or within 3 h after harvesting (*A. arguta* from wild populations) and ground to a powder in liquid nitrogen with a mortar and pestle. The powder was suspended in a 20-fold (w/v) organic solvent cocktail of 2:1 chloroform-methanol (v/v).

After the residues were removed with filter paper (Advantec qualitative no. 2; Toyo Roshi Kaisha Ltd., Tokyo, Japan), the filtrate was dried in a rotary evaporator (N-1200A; EYELA, Tokyo, Japan). The extracts were stocked at a concentration of 1 g wet weight per milliliter of *n*-hexane at 4°C until subsequent experiments.

#### Dry treatment of silver vine leaves

Twenty grams of fresh and undamaged green leaves of silver vine harvested from one branch in a wild population in Takizawa, Iwate Prefecture (39°48′16″N 141°06′17″E) on 31 August, 2022, were subjected to four treatments: fresh (undried) and 1 day, 4 days, and 7 days of drying. For the drying treatment, leaves were stored at 24 ± 2°C and 40 ± 3% humidity under atmospheric conditions. After complete drying, the leaves were weighed and organic solvent extracts were prepared as described above. The leaf extracts were dissolved in *n*-hexane to a concentration of 1 g wet leaves per milliliter.

#### Quantitative analysis of plant extracts by gas chromatography/mass spectrometry

All plant extracts were analyzed on a QP-2010 Ultra GC/MS (Shimadzu Co., Kyoto, Japan) operated in electron impact mode (70 eV) at an ion-source temperature of 200°C. The sample was injected into the injector at 250°C in splitless mode using an AOC-20i/s autosampler (Shimadzu Co.) in triplicate. The samples were separated on an InertCap Pure-WAX column (60 m × 0.25 mm internal diameter, 0.25 μm film thickness; GL Science, Tokyo, Japan). The GC was operated with helium as the carrier gas and a column flow rate of 1.66 mL/min. The GC temperature was maintained at 40°C for 2 min, increased to 250 °C at a rate of 4 °C/min, and held at 250°C for 20 min. GCMSsolution software (version 4.53; Shimadzu Co.) was used to process the raw data, identify the peaks from the total ion chromatograms, calculate the signal-noise ratio, and measure peak area. The contents of nepetalactol, nepetalactone, iridomyrmecin, isoiridomyrmecin, dihydronepetalactone, and isodihydronepetalactone, were quantified by the areas of the *m/z* 135, 81, 95, 81, 81, and 113 peaks, respectively, extracted from the full-scan data. The limit of detection (LOD) and the lower limit of quantification (LLOQ) were defined as S/N ratio ≥3 and 10, respectively. Calibration curves for nepetalactol and other iridoids were generated using 1.1, 2.2, 8.4, 34.2, 136.8, and 546.0 μg/mL and using 0.18, 0.36, 1.4, 5.7, 22.8, and 91.0 μg/mL, respectively.

#### Behavioral assays

The cats were not exposed to any olfactory stimulations at least 20 h prior to each assay and were placed in individual test cages (93 × 63 × 59 cm) for a few minutes to settle before each assay. The behavioral response of each cat was recorded using a digital video camera (Handycam HDR-CX680; Sony, Tokyo, Japan) placed in front of the cage. All of the movies of behavioral data were observed by R.U. and M.M. The duration and timing of the iridoid response to leaves or plant extracts (licking, chewing, rubbing, and rolling), as well as sniffing, touching the stimulant, walking, sitting, grooming, lying, or sleeping, were blindly assessed by R.U. and M.M. using Behavioral Observation Research Interactive Software (BORIS) version 8.9.11.^71^ Since the inter-rater agreement between R.U. and M.M. for the duration of the iridoid response was ranged from 71 to 92%, data obtained by R.U. was used for statistical analysis. In this paper, duration only of licking, chewing, rubbing, and rolling are counted as the part of the iridoid response because other behaviors such as sniffing are often observed against not only stimulus with iridoids but also other objects, for example toys, the anogenital region and other areas of another cat, a urine mark of other cats.^15^^,^^72^^,^^73^^,^^74^

Experiment 1 examined the behavioral responses of 10 cats when presented with silver vine extract continuously for 4 h. Four pieces of filter paper (4 × 4 cm, Advantec No. 526; Toyo Roshi Kaisha Ltd.) were each impregnated with 100 μL of extract of silver vine leaves that had been dried for 7 days (corresponding to 400 mg wet leaves in total). Each piece of filter paper contained approximately 3.2 μg iridoids with a nepetalactol:other iridoids ratio of about 1:1. Each filter paper was attached to the bottom of a Petri dish (9 cm diameter; AS ONE, Osaka, Japan), and the four dishes were fixed to the floor of a cage to form a 30 cm square rectangle using gummed-cloth tape. Separate bouts of feline response were defined as starting from the first iridoid response behavior aimed at the stimulus papers (rubbing, rolling, licking, or chewing) and ended after the last response behavior when this was followed by at least 5 min when there was no further contact with stimulus papers. We did not count a bout if cats licked and chewed the papers but did not rub against or roll on them.

Experiment 2 compared the responsiveness of eight cats toward extracts of fresh green leaves of silver vine and those of leaves that had been dried for 7 days. A 100 μL aliquot of each extract (corresponding to 100 mg wet leaf, iridoid contents: 7.5 μg in fresh leaves, 3.2 μg in dried leaves) was pipetted onto the bottom of separate Petri dishes (9 cm diameter; AS ONE) that had been rubbed with 40-grit sandpaper before pipetting. Each cat was presented with two dishes (fresh leaf extract versus 7-day-dried leaf extract) fixed 30 cm apart on the cage floor using gummed cloth tape until the cat showed disinterest in the stimuli for at least 10 min. The location of each dish on the right or left side of the cage floor was randomised.

Experiment 3 compared the responsiveness of six cats toward silver vine, *A. arguta*, and *A. kolomikta*. Fresh and undamaged green leaves of silver vine (wild, sex-unknown), *A. arguta* (wild, female), and *A. kolomikta* (garden, sex-unknown) were obtained as described above and presented to the cats on separate days. Approximately 5 g samples were placed on the cage floor within 4 h of sampling. As no subject showed much interest in *A. arguta* or *A*. *kolomikta*, we presented 5 g fresh green leaves of silver vine as the positive control immediately after these plant assays and confirmed the iridoid response to silver vine.

Experiment 4 examined the responsiveness of six cats to an extract of the roots of *A. deliciosa* (cultivar ‘Heyward,’ female). A 100 μL aliquot of the extract (corresponding to 100 mg wet leaves) and the control solvent *n*-hexane were pipetted onto separate Petri dishes, and the cats were tested as described for Experiment 2.

#### Quantification of serum cortisol and glucose

Blood samples were collected from the cephalic vein of 14 cats using a 23-gauge needle between 10:30 a.m. and 12:00 p.m. on day 1. The cats had not been stimulated by any odorants or toys on the day of sampling as a control condition. Three days later, these cats were presented with a Petri dish containing 100 μL of extract of silver vine leaves that had been dried for 7 days prepared as described for Experiment 2 to elicit the iridoid response. Immediately after the response, blood samples were taken from the cats as described above. On both days, cats were fed their morning food after the blood sampling. Serum samples were stored at −80°C until analysis.

Serum levels of cortisol were measured at a commercial laboratory (Fujifilm VET Systems, Tokyo, Japan) using the chemiluminescence immunoassay method. In cortisol assay, intra-assay precision values were the coefficient of variation (CV) 3.3%, 3.9%, and 3.8% in low, middle, and high concentrations, respectively. Inter-assay precision values were 5.3% and 3.8% in low and high concentrations, respectively. The lower limit of detection of cortisol was 0.14 μg/dL. Serum levels of glucose were measured using LabAssay Glucose (Fujifilm Wako Pure Chemical Co.).

#### Quantification of serum biomarkers of hepatic and renal injuries

Serum levels of alanine aminotransferase (ALT) and creatinine were monitored at most for approximately 3.2 years in thirteen cats that had responded (rubbed or rolled against a stimulus) when presented with silver vine or catnip, extracts of these plants, a synthesised iridoid, or a cocktail of the iridoids in test cages with 0.50–0.59 m^2^. The serum samples were obtained as described above and the levels of ALT and serum creatinine were analyzed using L-Type ALT (Fujifilm Wako Pure Chemical Co.) and LabAssay Creatinine (Fujifilm Wako Pure Chemical Co.), respectively. In all, 21 blood tests were conducted. We recorded the total number of responses that the subject had shown to iridoids up to each blood sampling time point, and the number of days over which cats experienced iridoid responses at each time point.

Serum levels of symmetric dimethylarginine (SDMA) were measured at a commercial laboratory (IDEXX Laboratories, Inc., Tokyo, Japan).

### Quantification and statistical analysis

All statistical analyses were performed with SPSS (version 29.0.0.0; IBM Corp., Armonk, NY, USA), R (version 4.1.3, The R Foundation for Statistical Computing, Vienna, Austria), and JMP software (version 12.0.0; SAS Institute Inc., Cary, NC, USA). All raw data are provided in the Data S1. QQ normal plots and Shapiro-Wilks tests confirmed that the values approximated normality where parametric analyses were used.

The non-parametric Wilcoxon matched-pair signed rank test (two-tailed, exact p-value was calculated if *N* < 10) was used to compare the relative durations of contact with the extract as assessed according to the iridoid response (licking, chewing, rubbing, and rolling), sniffing, and touching versus those of other behaviors (walking, sitting, grooming, lying, and sleeping). Similar tests were used to compare the durations of the first response bout toward the extract versus those of the second response bout, the response durations to extracts of fresh versus those of dried leaves, and response durations to the extract of *A. deliciosa* root versus those of the control stimulus.

The duration of the response to 5 g leaves from silver vine, *A. arguta*, and *A. kolomikta* was compared using the Friedman test, with differences between pairs of groups tested by Steel-Dwass post hoc comparisons.

Serum cortisol and glucose levels were log-transformed to meet the assumptions for parametric analysis. The log values were compared between control conditions and after the response to the silver vine extract using a paired t-test.

Serum ALT and creatinine values were log-transformed. A linear mixed effects model (using the lme4 package in R^75^) was used to examine the fixed effects of the total number of iridoid responses and the numbers of days over which cats experienced iridoid response on log values of ALT and creatinine with subject cat as the random effect.

### Additional resources

No additional resources were used in this study.

## Data Availability

• All source data to generate all the figures are included in the Data S1. • This paper does not report original code. • Any additional information required to reanalyze the data reported in this paper is available from the lead contact upon request.

## Supplemental information

Document S1. Figure S1 and Table S1

Data S1. All of the raw data in this study, related to all of Figures and Tables
