# Supplementary material for: Assessing the safety and suitability of using silver vine as an olfactory enrichment for cats
Source: iScience. 2023 Sep 7;26(10):107848. doi: 10.1016/j.isci.2023.107848 (PMC10558724; doi:10.1016/j.isci.2023.107848)
Supplement: Document S1. Figure S1 and Table S1 [file mmc1.pdf]

iScience, Volume *26*

Supplemental information

Assessing the safety and suitability of using silver vine as an olfactory enrichment for cats

Reiko Uenoyama, Sae Ooka, Tamako Miyazaki, Hiroki Mizumoto, Toshio Nishikawa, Jane L. Hurst, and Masao Miyazaki

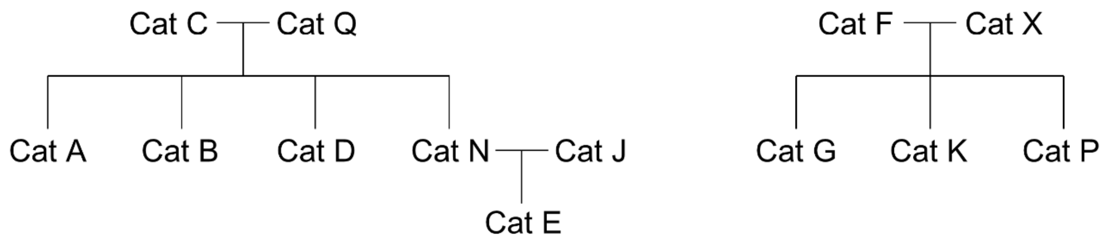

Figure S1. Kinship of subject cats, Related to Figure 1.

Family trees showing blood relationships between subject cats. Cat X did not participate in this study.

Table S1. The histories of exposure to iridoids and serum SDMA levels in 12 cats, Related to Table 1.

Cat ID	Age (day)	Total number of iridoid response	Days over which response experienced	Serum SDMA (μg/dL)
Cat A	789	30	658	9
Cat B	789	60	739	14
Cat C	3585	117	1215	9
Cat D	789	37	658	10
Cat E	323	5	77	10
Cat F	3962	127	1215	12
Cat G	3459	48	1207	9
Cat J	868	15	341	9
Cat K	1539	36	1214	14
Cat M	1534	52	607	10
Cat P	1539	30	1207	8
Cat Q	1349	20	608	9
